# Leveraging Large Language Models for Decision Support in Personalized Oncology

**DOI:** 10.1001/jamanetworkopen.2023.43689

**Published:** 2023-11-17

**Authors:** Manuela Benary, Xing David Wang, Max Schmidt, Dominik Soll, Georg Hilfenhaus, Mani Nassir, Christian Sigler, Maren Knödler, Ulrich Keller, Dieter Beule, Ulrich Keilholz, Ulf Leser, Damian T. Rieke

**Affiliations:** 1 Charité Comprehensive Cancer Center, Charité–Universitätsmedizin Berlin, Corporate Member of Freie Universität Berlin and Humboldt-Universität zu Berlin, Berlin, Germany; 2 Core Unit Bioinformatics, Berlin Institute of Health at Charité–Universitätsmedizin Berlin, Charitéplatz 1, Berlin, Germany; 3 Knowledge Management in Bioinformatics, Humboldt-Universität zu Berlin, Berlin, Germany; 4 Department of Hematology, Oncology and Cancer Immunology, Campus Benjamin Franklin, Charité–Universitätsmedizin Berlin, Corporate Member of Freie Universität Berlin and Humboldt-Universität zu Berlin, Berlin, Germany; 5 Department of Endocrinology and Metabolic Diseases, Charité Universitätsmedizin Berlin, Department of Endocrinology and Metabolic Diseases, Corporate Member of Freie Universität Berlin and Humboldt-Universität zu Berlin, Berlin, Germany; 6 Department of Hematology, Oncology and Cancer Immunology, Campus Charité Mitte, Charité–Universitätsmedizin Berlin, Corporate Member of Freie Universität Berlin and Humboldt-Universität zu Berlin, Berlin, Germany; 7 German Cancer Consortium and German Cancer Research Center, Partner Site Berlin, Germany

## Abstract

**Importance:**

Clinical interpretation of complex biomarkers for precision oncology currently requires manual investigations of previous studies and databases. Conversational large language models (LLMs) might be beneficial as automated tools for assisting clinical decision-making.

**Objective:**

To assess performance and define their role using 4 recent LLMs as support tools for precision oncology.

**Design, Setting, and Participants:**

This diagnostic study examined 10 fictional cases of patients with advanced cancer with genetic alterations. Each case was submitted to 4 different LLMs (ChatGPT, Galactica, Perplexity, and BioMedLM) and 1 expert physician to identify personalized treatment options in 2023. Treatment options were masked and presented to a molecular tumor board (MTB), whose members rated the likelihood of a treatment option coming from an LLM on a scale from 0 to 10 (0, extremely unlikely; 10, extremely likely) and decided whether the treatment option was clinically useful.

**Main Outcomes and Measures:**

Number of treatment options, precision, recall, F1 score of LLMs compared with human experts, recognizability, and usefulness of recommendations.

**Results:**

For 10 fictional cancer patients (4 with lung cancer, 6 with other; median [IQR] 3.5 [3.0-4.8] molecular alterations per patient), a median (IQR) number of 4.0 (4.0-4.0) compared with 3.0 (3.0-5.0), 7.5 (4.3-9.8), 11.5 (7.8-13.0), and 13.0 (11.3-21.5) treatment options each was identified by the human expert and 4 LLMs, respectively. When considering the expert as a criterion standard, LLM-proposed treatment options reached F1 scores of 0.04, 0.17, 0.14, and 0.19 across all patients combined. Combining treatment options from different LLMs allowed a precision of 0.29 and a recall of 0.29 for an F1 score of 0.29. LLM-generated treatment options were recognized as AI-generated with a median (IQR) 7.5 (5.3-9.0) points in contrast to 2.0 (1.0-3.0) points for manually annotated cases. A crucial reason for identifying AI-generated treatment options was insufficient accompanying evidence. For each patient, at least 1 LLM generated a treatment option that was considered helpful by MTB members. Two unique useful treatment options (including 1 unique treatment strategy) were identified only by LLM.

**Conclusions and Relevance:**

In this diagnostic study, treatment options of LLMs in precision oncology did not reach the quality and credibility of human experts; however, they generated helpful ideas that might have complemented established procedures. Considering technological progress, LLMs could play an increasingly important role in assisting with screening and selecting relevant biomedical literature to support evidence-based, personalized treatment decisions.

## Key Points

**Question:**

Can current conversational large language models (LLMs) be used as a tool for personalized decision-making in precision oncology?

**Findings:**

In this diagnostic study, treatment option identification from 4 LLMs for 10 fictional patients deviated substantially from expert recommendations. Nevertheless, LLMs correctly identified several important treatment strategies and partly provided reasonable suggestions that were not easily found by experts.

**Meaning:**

These results suggest that LLMs are not yet applicable as a routine tool for aiding personalized clinical decision-making in oncology, but do improve upon existing LLM-based methods.

## Teaser

This diagnostic study evaluates treatment recommendations for 10 fictional patients with advanced cancer made by 4 large language models, as evaluated by members of a molecular tumor board.

## Introduction

Precision medicine describes the concept of personalized clinical decision-making by accounting for individual variation.^1^ This concept requires an evidence-based interpretation of variations as biomarkers. In oncology, the integration of genetic (tumor) alterations as predictive biomarkers comes closest to this concept of personalized medicine, as targeted treatment of well-defined molecular alterations shows clinical efficacy.^2,3,4^ Accordingly, comprehensive multigene sequencing has become a standard tool for diagnosis and treatment allocation and is used increasingly in multiple tumor types.^2,3,4^ However, the identification of uncommon and complex molecular alterations or defined biomarkers falling outside currently established guidelines and recommendations creates challenges for clinical decision-making. These findings are frequently discussed in specialized and interdisciplinary molecular tumor boards (MTB).^5^ Especially in these settings, the clinical interpretation of molecular alterations remains manual work based on search engines and specialized curated databases.^6^ Yet, these databases contain mostly nonoverlapping information,^7^ which provides strong evidence for their incompleteness. Accordingly, the selection and interpretation of evidence for less well-characterized molecular alterations create inter-interpreter heterogeneity.^8^

The development of new artificial intelligence systems (AI), such as large language models (LLMs),^9,10^ has improved the quality of automated analysis of large and complex data sets considerably. LLMs have already been assessed in various biomedical contexts, such as clinical language understanding^11^ and optimization of general clinical decision support.^12^ Their potential role in supporting personalized oncology remains undefined. Here, we present results from an explorative analysis of LLM-generated treatment recommendations to assist an MTB.

## Methods

This diagnostic study of the development of LLM-based treatment generation followed the Standards for Reporting of Diagnostic Accuracy (STARD) reporting guideline. An overview of the workflow and additional context is provided in eMethods and eFigure 3 in Supplement 1. Review by the Universitätsmedizin Berlin ethics review board was not required because no patient data were used.

### Development of Fictional Case Vignettes

We created molecular profiles for 10 fictional patients based on realistic clinical scenarios, similar to a previous study.^8^ Cases covered 7 different tumor entities and included 59 distinct molecular alterations largely falling outside current guidelines. An overview of all cases is available in the Table, and a detailed description is in eTable 1 in Supplement 1. Cases were designed to represent tumor types and alterations typically encountered in molecular tumor boards, including an overrepresentation of lung adenocarcinoma cases, where multigene sequencing is standard-of-care.^13^

**Table.** Patient Characteristics of Mock Patients in Analyzing of Artificial Intelligence Large Language Models

Variable	Participants, No. (%) (N = 10)
Age, median (IQR) [range], y	57 (48-59) [26-79]
Sex	
Female	3 (30)
Male	3 (30)
Unknown	4 (40)
Diagnosis	
Lung adenocarcinoma	4 (40)
Other	6 (60)
Tumor purity, median (IQR) [range], %	60 (50-77.5) [30-80]
Type of sequencing	
Panel	9 (90)
Whole exome sequencing	1 (10)
TMB, median (IQR) [range]	7.2 (3.2-11.1) [3.2-12.8]
Total variants, median (IQR) [range], No.	3.5 (3.0-4.75) [2.0-18.0]

Abbreviation: TMB, tumor mutational burden.

### Clinical Interpretation of Molecular Data

Each case vignette was assigned to 1 expert physician of the Charité MTB for manual clinical interpretation of molecular findings, following previously described workflows.^5^ Additionally, 4 different LLMs were tasked to generate treatment options: BioMed LM (MosaicML; Stanford University) (LLM 1),^14^ Perplexity.ai (University of California, Berkeley) (LLM 2),^15^ ChatGPT (OpenAI) (LLM 3),^16^ and Galactica (Meta) (LLM 4).^17^ These 4 were selected to compare across 4 different criteria: type of usage (local installation vs online, important regarding data privacy requirements), model size (in terms of computational resources required), openness (whether an integrated retrieval engine is used, impact on up-to-datedness), and pretraining domain (general or medical, impact on result quality) (eTable 2 in Supplement 1).

Because the field of LLMs is rapidly evolving, we also performed a poststudy comparison with ChatGPT version 4, which was not available at the time of the study with MTB members. Complete information regarding the fictional patient cases, the LLM-generated answers, and the scripts to generate the results are available online.^18^

### Prompting LLMs

By explorative analysis, we first designed a general natural language prompting template that we then adapted specifically to the LLM at hand (eTable 3; eFigure 1 in Supplement 1). These adapted templates were then used for prompting an LLM for each patient case separately. An exemplary template used as an LLM prompt was: “Given a <*diagnosis of disease>* with mutations <*enumeration of mutations>*. What are possible targeted therapies available? Please always add NCT/PubMed IDs and specify evidence level and clinical significance if possible.” The bracketed slots were replaced for each patient case with a diagnosis and listed molecular alterations. For example, in case 10 the diagnosis of disease was listed as *lung adenocarcinoma* and the enumeration of mutations as EGFR E746_A750del, EGFR C797S, and STK11 C210*.

We gathered all answers generated by the LLM and transformed them into a unified table with each row containing the molecular alteration examined, the LLM used, their recommended treatment option plus additional information, in particular with references (ie, clinicaltrials.gov National Clinical Trial [NCT] or PubMed identifier), mechanism of proposed drugs, evidence level of the corresponding study, phase (I, II, or III), evidence for drug efficacy evaluation (eFigure 2 in Supplement 1). This information was directly extracted from the LLM answer and not checked for accuracy at this point. The structured summary enabled comparison of results across LLMs and with the manual annotations from human experts.

### Assessment of Results in an Interdisciplinary MTB

Treatment options from the 4 LLMs were condensed into 2 types of summaries for evaluation in the MTB: (1) combined treatment option, which contained options identified by at least 2 different LLMs; and (2) clinical treatment option, containing options with at least 1 associated NCT or PubMed identifier. These 2 lists and a third, manually annotated list of treatment options were masked and presented to the MTB at Charité’s Comprehensive Cancer Center.

We created an online survey for MTB members (eTable 4 in Supplement 1). Participants rated the likelihood of a treatment option coming from an AI (on a scale from 0 to 10, with 10 signifying options most likely coming from AI). Furthermore, the MTB members selected which option they would most likely pursue further and rated the general usefulness of recommendations.

### Statistical Analysis

The concordance of LLM-generated treatment options (4 individual and 2 combined options) with the manually generated treatment options as criterion standard was measured using precision, recall, and F1 score. Precision, which denotes the fraction of relevant treatment options among the suggested options, was defined as precision = true positives / (true positives + false positives). Recall, or the fraction of all treatment options in the criterion standard found by LLMs, was defined as recall = true positives / (true positives + false negatives). The F1 score is the harmonic mean of precision and recall, and thus penalizes unbalanced precision and recall scores (ie, is higher when both precision and recall have similar values): F1 score = (2 × precision × recall) / (precision + recall). The higher any of the 3 scores, the better the LLM has performed compared with the human recommendations, with 1 being the maximum value for each score.

Data analysis, calculation of precision, recall and F1 scores were done in R version 4.3.0 (R Project for Statistical Computing). All scripts and raw data are available in the study github repository.^18^

## Results

### Quantitative Evaluation of Treatment Options

**Figure 1.**
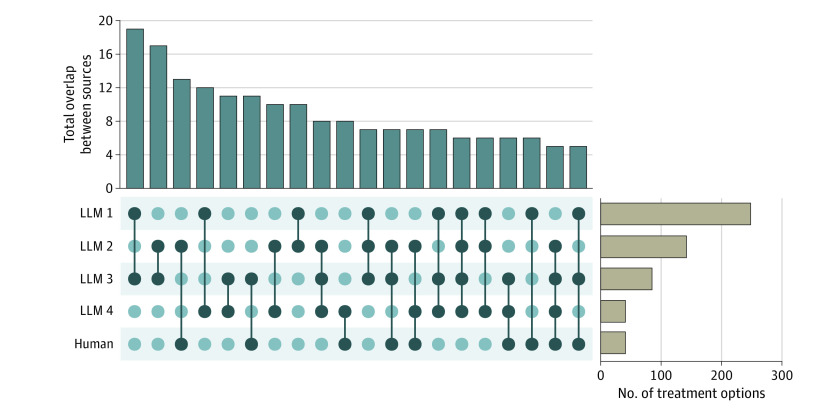
Overlap Analysis of Treatment Options Total number of recommendations (right-hand bar plot) and overlap between the recommended treatment options from the different large language models (LLMs) and a human annotator given 58 unique alterations across 10 fictional patients. Sources under comparison are indicated in the matrix (indicated by dark dots) and number of treatment options coming from multiple sources are shown in the upper bar plot. For clarity, only overlaps with 5 or more treatment options are shown.

Ten fictional cancer patients (4 with lung cancer, 6 with other cancer types) with a median (IQR) of 3.5 (3.0-4.8) molecular alterations (59 molecular alterations total) were designed and submitted to an expert physician and 4 LLMs to identify treatment options (Table; eTable 1 in Supplement 1). Expert interpretation identified treatment options for 21 of the 59 molecular alterations with a median of 2 unique treatment options per alteration (range, 1-4 treatment options). The number of alterations with a treatment option identified by the LLMs was 54 for LLM 1, 38 for LLM 2, 37 for LLM 3, and 24 for LLM 4. A median (IQR) of 4.0 (3.0-5.0) treatment options per alteration were identified by LLM 1, 3.0 (2.0-5.0) by LLM 2, 2. 0 (1.0-2.0) by LLM 3, and 1.0 (1.0-1.3) by LLM 4. These numbers corresponded to a median number of 4 treatment options per patient for expert curation, 13.0 (11.3-21.5) for LLM 1, 11.5 (7.8-13.0) for LLM 2, 7.5 (4.3-9.8) for LLM 3, and 3.0 (3.0-5.0) for LLM 4. The highest absolute overlap in treatment options between 2 LLMs was 19 (LLMs 1 and 3) (Figure 1).

**Figure 2.**
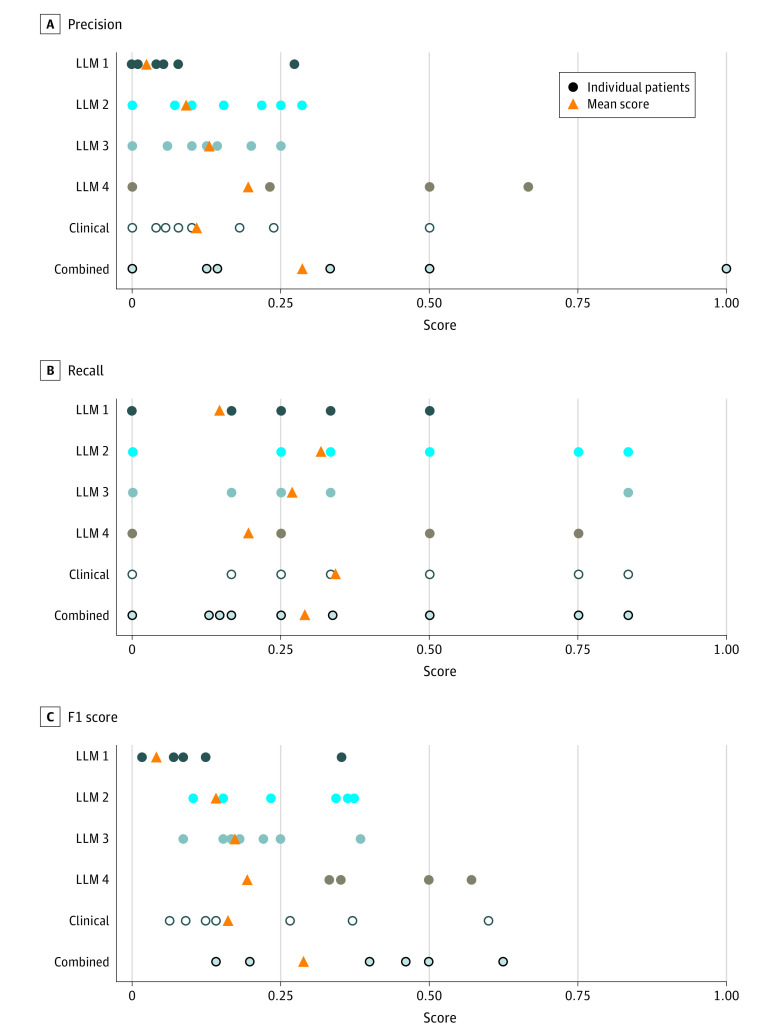
Quantitative Analysis of Model Performance

When manually identified treatment options were set as the criterion standard, the LLMs reached F1 scores of 0.04 (LLM 1), 0.14 (LLM 2), 0.17 (LLM 3), and 0.19 (LLM 4) (Figure 2). Because of the limited individual performance, LLM-generated treatment options were summarized into combined treatment option and clinical treatment option for further analyses. Combined treatment option considered only treatment options identified by more than 1 LLM and clinical treatment option were restricted to treatment options associated with a concrete (although possibly wrong) reference to clinical evidence. Combined treatment options reached an F1 score of 0.29, thus outperforming the best individual performance of an LLM. The clinical treatment options reached the highest recall of 0.34 of all the LLMs.

### Qualitative Analysis

**Figure 3.**
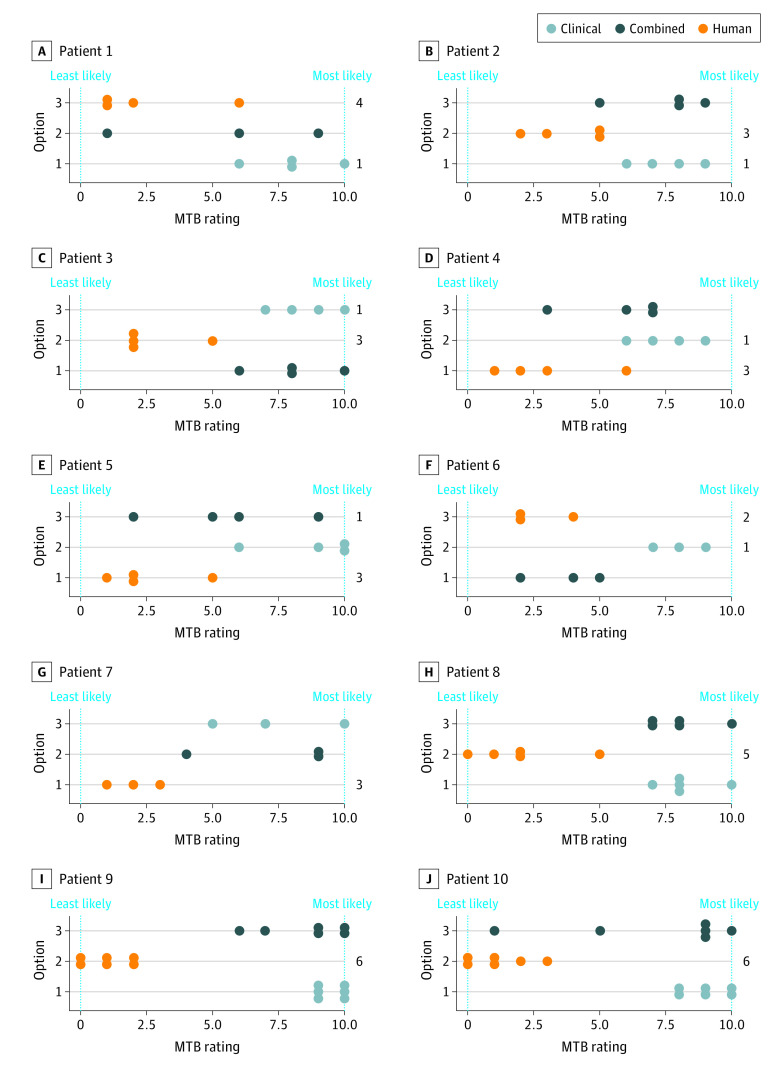
Treatment Evaluations of 10 Fictional Patients by Molecular Tumor Board (MTB) Experts For each patient, 3 options for treatment recommendations were presented to the MTB. Members of the MTB ranked each option from 0 (least likely to come from a large language model [LLM]) to 10 (most likely to come from an LLM). In addition, the totals on the right side of the plot indicate how many participants would choose the given option for the patient.

The set of all obtained treatment options was masked and presented to an interdisciplinary MTB. MTB members were asked to rate the likelihood of treatment options coming from an LLM (on a 10-point scale, with 0 being extremely unlikely and 10 extremely likely) and their clinical usefulness. LLM-generated combined treatment options and clinical treatment options yielded median (IQR) scores of 7.5 (5.3-9.0) and 8.0 (7.5-9.5) points, respectively. Manually generated options reached a median score of 2.0 (1.0-3.0) points. Thus, MTB members were able to identify LLM-generated treatment options with high confidence (Figure 3).

In the 43 overall answers, MTB participants further indicated which 1 of the 3 treatment options they would most likely consider for clinical decision-making. In 37 cases, they preferred to pursue the human annotation, and in 6 cases, they indicated a preference for an LLM-generated treatment option (Figure 3). At least 1 LLM-generated treatment option per patient was considered helpful by MTB members. The accuracy of provided references was frequently cited as a reason why LLM-generated treatment options were disregarded. LLMs 1 and 4 were not able to provide any useful references in our preliminary studies, so prompting for references was eventually stopped for both. LLM 3 provided 85 unique NCT identifiers across 74 of its 86 treatment options. LLM 2 was able to provide references for 131 of its 142 treatment options, 34 of them being unique NCT identifiers and the rest being PubMed and PubMed Central identifiers or other web resources. We performed an assessment to check how many of the suggested references, specifically the NCT identifiers, linked to an existing study. Out of the 85 provided NCT identifiers by LLM 3, 27 did not exist. In contrast, none of the 34 NCT identifiers provided by LLM 2 were hallucinated (eFigure 4 in Supplement 1).

### Unique Treatment Recommendations

Although the treatment options presented by LLMs did not match all recommendations from expert human annotators, 2 unique treatment options (including 1 unique treatment strategy) were pointed out as clinically useful by MTB members that were identified only by the LLMs and not by the human expert. The unique treatment strategy was antiandrogen therapy in a patient with salivary duct carcinoma with *HRAS* and *PIK3CA* variation. *HRAS* and *PIK3CA* comutated salivary duct carcinoma usually stain positive for the androgen receptor in immunohistochemistry.^19^ Antiandrogen therapy was not suggested by the human expert because no immunohistochemistry results were provided.

### Retrospective Analysis of an Updated LLM

To evaluate how newer models of AI assistance may affect results, we retrospectively analyzed differences in results from ChatGPT 3 with those of its most recent version, which was not available at the time of the primary analysis. The newer version generated 74 treatment options for the 10 fictional patients, compared with the 85 treatment options from ChatGPT 3. Twenty-six treatment options overlapped between both versions, showing the high instability of updated models. In comparison with the human expert, the updated LLM reached an F1 score of 0.26, surpassing all 4 LLMs we tested in the study (eFigure 5 in Supplement 1). In contrast to ChatGPT 3, its newer version reduced the number of hallucinated references: only 1 out of 17 unique NCT identifiers provided did not exist vs 27 out of 85 with ChatGPT 3.

## Discussion

Artificial intelligence systems are used increasingly for health care applications.^20^ Previous reports have shown good performance for well-defined tasks in radiology, dermatology, or pathology.^21^ More recently developed LLMs might also help to deal with more complex tasks in medicine, such as clinical decision-support in organ-specific tumor boards to facilitate the implementation of existing guidelines.^22,23,24^ Integrating multidimensional data beyond established guidelines is an additional challenge typically faced in precision oncology and molecular tumor boards, making this a compelling use case for LLMs. This study reports results from an analysis of LLM-supported decision-making to facilitate personalized oncology. Despite the small sample size of 10 fictional patients, we were able to generate first results for model performance that overall were consistent across LLMs.

The F1 scores reached by LLMs compared with expert recommendations were generally low (below 0.3). The best-performing LLM generated a recall value of 0.34. This result suggests that applying LLMs to prefilter treatment options for human experts is not yet efficient, as important recommendations were not reported. However, these results came close to the performance of established precision oncology knowledge databases (eFigure 5 in Supplement 1). Additionally, such an interpretation considers single-expert annotation as criterion standard, despite considerable inter-interpreter heterogeneity.^6^ Furthermore, at least 1 LLM-generated treatment option per patient was considered practically relevant, and 2 treatment options were identified only by an LLM suggesting their potential usefulness as a complementary search tool.

A comparison of the 4 examined LLMs shows that the smaller model BioMed LM trained only on PubMed did not reach the performance of the 3 larger general purpose LLMs trained on further corpora. This is consistent with previous results suggesting that an increase in model size is one of the key factors for improving performance.^25,26^ The F1 scores for extracted treatment options were similar across the 3 larger models. However, for MTB members, the quality of the provided study references was decisive for their assessment that most LLM-generated treatment options were not actionable. Analyses of LLMs for other complex medical tasks observed similar challenges.^27,28^ Future developments thus should focus on identifying adequate references for supporting recommendations.

Other specific requirements exist for health care applications of LLMs. Online-only models like ChatGPT and Perplexity.ai allow for a low-maintenance integration in existing workflows and provide continuous updates but require disclosing patient data to commercial services. Uncontrolled model updates furthermore make the quality of results unpredictable and destroy reproducibility of recommendations. On the other hand, stand-alone applications like BioMed LM or Galactica require local installation and maintenance but have the advantage of full data privacy and reproducibility of results. Updates can be performed in a controlled manner and follow an internal versioning control for ensuring accountability of recommendations. Selecting the most suitable tool for specific requirements therefore needs careful prior evaluation. Selection of LLMs is additionally complicated by the rapid development of the field, with new LLMs being published at an almost weekly basis.^29,30^ From a conceptual point of view, they rely on the same computational model as ChatGPT, but use different training corpora, different inference architecture, and different training procedures. Being up-to-date thus requires continuous repetition of assessments with new models. In a retrospective analysis, a newer model of ChatGPT reached a higher F1 score than the 4 LLMs included in the primary analysis and reduced the number of hallucinated references in comparison with its predecessor. This comparison highlights that the performance of LLMs is highly influenced by versioning, and rapid improvements are expected in the future.

The integration of complex clinical and molecular data by LLMs, as shown here for precision oncology, also holds important implications for other fields in oncology and medicine. For example, an automated and comprehensive review of existing data could help design clinical and preclinical research.^31^ This approach could be especially useful in precision oncology, where a highly individual combination of biomarkers limits traditional trial design.^32^

### Limitations

This study had several limitations. The limited number of fictional patients, as well as the rapid development of new LLM models and versions, limit conclusions from study results. The design of highly dimensional fictional patients and an analysis plan including 4 different LLM with different technological backgrounds still allowed for a first validation of LLM for precision oncology applications.

## Conclusions

In this diagnostic study of LLM-based decision support for personalized oncology, LLMs were not yet suitable to automate an MTB annotation process. However, rapid developments can be expected in the near future and LLMs could already be used to complement the screening of large biomedical data sets. Addressing the accountability of clinical evidence, data privacy and quality control remain key challenges.

## Notes

Supplement 1.
eMethods.
**eTable 1.** Detailed Descriptions of the 10 Mock Patients **eTable 2.** Comparison of the LLMs Used in This Study **eTable 3.** Prompt Templates for All LLMs in the Given Study **eTable 4.** Questions for the Survey **eFigure 1.** Number of Treatment Options per Prompt Type **eFigure 2.** Workflow of LLM Prompting **eFigure 3.** General Workflow of the Analysis **eFigure 4.** Number of Unique Clinical Trials Suggested by LLMs and the Oncological Experts **eFigure 5.** Precision, Recall, and F1 Scores for the Structured Databases and LLMs Compared With the Human Criterion Standard
eReferences.

Supplement 2.
Data Sharing Statement
