# Supplementary material for: Leveraging Large Language Models for Decision Support in Personalized Oncology
Source: JAMA Netw Open. 2023 Nov 17;6(11):e2343689. doi: 10.1001/jamanetworkopen.2023.43689 (PMC10656647; doi:10.1001/jamanetworkopen.2023.43689)
Supplement: Supplement 1. — eMethods. eTable 1. Detailed Descriptions of the 10 Mock Patients eTable 2. Comparison of the LLMs Used in This Study eTable 3. Prompt Templates for All LLMs in the Given Study eTable 4. Questions for the Survey eFigure 1. Number of Treatment Options per Prompt Type eFigure 2. Workflow of LLM Prompting eFigure 3. General Workflow of the Analysis eFigure 4. Number of Unique Clinical Trials Suggested by LLMs and the Oncological Experts eFigure 5. Precision, Recall, and F1 Scores for the Structured Databases and LLMs Compared With the Human Criterion Standard eReferences. [file jamanetwopen-e2343689-s001.pdf]

Supplemental Online Content

Benary M, Wang XD, Schmidt M, et al. Leveraging large language models for decision support in personalized oncology. *JAMA Netw Open.* 2023;6(11):e2343689. doi:10.1001/jamanetworkopen.2023.43689

eMethods.

eTable 1. Detailed Descriptions of the 10 Mock Patients

eTable 2. Comparison of the LLMs Used in This Study

eTable 3. Prompt Templates for All LLMs in the Given Study

eTable 4. Questions for the Survey

eFigure 1. Number of Treatment Options per Prompt Type

eFigure 2. Workflow of LLM Prompting

eFigure 3. General Workflow of the Analysis

eFigure 4. Number of Unique Clinical Trials Suggested by LLMs and the Oncological Experts

eFigure 5. Precision, Recall, and F1 Scores for the Structured Databases and LLMs Compared With the Human Criterion Standard

eReferences.

This supplemental material has been provided by the authors to give readers additional information about their work.

© 2023 Benary M et al. *JAMA Network Open.*

eMethods

Patient characteristics

The ten fictional patients were created based on realistic clinical scenarios as reported previously [1]. In total, 58 distinct molecular alterations were integrated (details in eTable 1).

Comparison of Large Language Models

We included four large language models (LLMs) to assess similarities and differences between commonly used models. The availability of a model is an important aspect to ensure its widespread usage. A model accessible only via a web page (ChatGPT and Perplexity.ai) is simple to navigate and integrated into the annotation workflow of a clinician. A stand-alone application may be more cumbersome to set up (BioMed LM and Galactica). However, a stand-alone local installation has a significant advantage regarding data privacy: Clinical patient data is often highly sensitive, so it should not be shared with unauthorized third parties like online LLM providers.

The underlying model and its number of parameters determine how much knowledge a model can store internally. The higher the number of parameters, the better a model usually performs in knowledge-intensive tasks like question answering [2]. We examined medium-sized domain-focused language models like BioMed LM pre-trained on PubMed, large general-purpose language models like ChatGPT and compared their expressivity.

A summary of each characteristic for the LLMs is found in eTable 2.

Prompting Large Language Models

Choosing appropriate prompts goes a long way in getting helpful answers from an LLM. We experimented with different kinds of prompts for each LLM. Depending on the prompt, the numbers of proposed treatment options may vary greatly, as was in the case of BioMed LM: Prompting the model with an open question like "*What drugs are there for treatment of variant PIK3CA E545K in oropharyngeal carcinoma?"* resulted in one TO on average whereas prompting it with completing part of a sentence like "*Targeted therapies for oropharyngeal carcinoma with a mutation in PIK3CA E545K include inhibitors like ..."* resulted in an average of three TOs per alteration (**eFigure 1**). The list of all prompts is available in

Survey for evaluating treatment options

We created a survey to address two types of questions:

1. Are clinicians able to distinguish between treatment options from an LLM or an expert physician?
2. Which treatment options would the participants further pursue and rated their usefulness?

The survey was anonymous, but the participants provided information on their profession and experience in molecular tumor boards. For each patient, we provide the slides discussed in the molecular tumor board for reference. The specific questions of the survey are in eTable 3.

Comparison to curated databases

An alternative way to reliably retrieve relevant evidence from the biomedical literature is using structured databases like CiVIC[4] or OncoKB[5]. They consist of high-quality data curated manually by expert oncologists. Yet, these databases contain mostly non-overlapping literature, which requires querying several databases or the development of harmonized meta-databases[6]. In an updated analysis of databases for this manuscript, we calculated precision, recall, and F1-Score against the human gold standard and compared them to the LLMs.

For reference, we included all treatment options from the two knowledge bases if they are supporting in treating a given alteration in at least one study independent of the evidence level of the study. OncoKB achieved a recall of 0.43 and an F1-score of 0.32, thus outperforming the best annotations of the LLMs. In contrast, CiVIC achieves a recall of 0.14 and an F1-score of 0.13 (eFigure 4). These heterogeneous results underline the difficulty of annotating variants and may indicate inter-annotator differences for the gold standard.

eTable 1. Detailed Descriptions of the 10 Mock Patients

Age	Sex	Diagnosis	Stage	Type of Sequencing	Molecular Alterations
56		Lung Adenocarcinom a	IV	Panel	KRAS p.G13D, TP53 p.A276G, PTPRS R238*, ZFHX3 p.F2994L, CDH1 p.D433N
26		Urachal Carcinoma	IV	Panel	KRAS p.G12V, BCORL p.R1332*, TP53 p.H214f s*7, CDKN2C p.L65F, MAP3K1 p.T949_E950ins T, MYCN p.E47fs*8, CTNNA1 p.K577_L578 > T KL, JAK1 p.I597M, FANCL p.T367fs*12+, PIK3C A amplification (n>6), MYC amplification (n>6), MYCL1 amplification (n>6), SOX2 amplification (n>6), MUTYH amplification (n>6)
58		Thymic adenomcarcino ma	IV	Whole-Exome	Germline: BRCA2 p.K3326* (1N), Tumor: SMAD 4 p.C363R (1N), TP53 p.305fs (2N_LOH), CDK N1B p.K100N (2N_LOH), ATM p.E1666* (4N), MAP3K8 p.H236Y (1N), TRAF1 p.R70H (2N), H DAC2 p.R409* (1N), TMEM111-TDRD3 fusion, PRKDC-CDH17 fusion, EXT1-MAGI2 fusion, ER BB2 RNA overexpression (RPKM tumor 45 v 1.8 control), ERBB3 RNA overexpression (RPKM tu mor 65.9 v 0.2 control), PDGFRB RNA overexpr ession (RPKM tumor 35.8 vs 2.3 control), TGFA RNA overexpression (RPKM tumor 14.2 v 0.4 co ntrol, EGF RNA overexpression (RPKM tumor 1. 9 vs 0.1 control), FGFR3 RNA overexpression (RPKM tumor 11.4 vs 1.9 control), MET RNA ove rexpression (RPKM tumor 22.1 v 1.4 control)
59		Oropharyngeal carcinoma	IV	Panel (Tumor Purity 60%)	PIK3CA p.E545K (AF 25%), MAPK1 p.E322K (A F 10%), FGFR3 p.D786N (AF 30%)
64	w	Lung Adenocarcinom a	IV	Panel (Tumor Purity 30%), TMB 3.8 Mut/Mb	EGFR p.E746_A750del (AF 43%), TP53 p.A138 _Q144del (AF 37%), MET Amplification FISH po sitive
59	m	Lung Adenocarcinom a	IV	Panel (Tumor Purity 40%)	KRAS p.G12C (AF 18%), KEAP1 p.L276F (AF 4 5%), STK11 p.K83Tfs*13 (AF 38%)
41	w	Melanoma	IV	Panel (Tumor Purity 80%), TMB 12.8 Mut/Mb	NF1 p.I1605fs (AF 39%), TP53 c.672+1G>A (AF 50%), RB1 p.Q846* (AF 20%), TERT p.R859Q (AF 41%)
46	m	Cholangiocarcin oma	IV	Panel (Tumor Purity 80%), TMB 1.2 Mut/Mb	FGFR2::BICC1 Fusion, TP53 p.E258* (AF 52%)
79	m	Salivary Duct Carcinoma	IV	Panel (Tumor Purity 75%), TMB 10.5 Mut/Mb	HRAS p.Q61R (AF 44%), PIK3CA p.E545K (AF 39%), TP53 p.T211A (AF 60%), KMT2C p.P249 3Q (AF 21%)
52	w	Lung Adenocarcinom a	IV	Panel (Tumor Purity 60%)	EGFR p.E746_A750del (AF 50%), EGFR p.C79 7S (AF 29%), STK11 p.C210* (AF 39%)

© 2023 Benary M et al. *JAMA Network Open.*

eTable 2. Comparison of the LLMs Used in This Study

	ChatGPT	Perplexity.ai	BioMed LM	Galactica
**Availability**	Online	Online	Local	Local
**Parameter Size**	175B	175B	2.7B	30B
**Underlying Model**	GPT 3.5	GPT 3.5	GPT 2	Galactica large
**Training Corpus**	Not available	Not available	PubMed	Scientific texts (PubMed, arXive, etc), Reference material (Wikipedia, StackExchange), Knowledge bases (Uniprot, Reactome)
**Access to search engine**	No	Yes	No	No

© 2023 Benary M et al. *JAMA Network Open.*

eTable 3. Prompt Templates for All LLMs in the Given Study

	**Question type**	**Prompt template**
BioMed LM P1	Closed question	Are there any drugs for treatment of the variant <MUTATION> in <DISEASE>?
BioMed LM P2	Open question	What drugs are there for treatment of variant <MUTATION> in <DISEASE>?
BioMed LM P3	List completion	Targeted therapies for <DISEASE> with a mutation in <MUTATION> include possible inhibitors like …
BioMed LM P4	Sentence completion	A possible treatment for <DISEASE> with a mutation in <MUTATION> are …
Perplexity.ai P1	Open question (General treatments)	Targeted therapy for <MUTATION> mutation in <DISEASE>?
Perplexity.ai P2	Open question (Clinical trials)	Fitting clinical trials for <MUTATION> mutation in <DISEASE>?
ChatGPT	Open question	Given a <DISEASE> with <MUTATION 1>, <MUTATION 2>, etc. mutations. What are possible targeted treatments available? Please always add NCT/PubMed IDs and specify evidence level and clinical significance if possible.
Galactica P1	Open question	What are treatment options for <DISEASE> patients with a mutation in <MUTATION>?
Galactica P2	Open question (Rephrased)	What are treatment options for <DISEASE> patients with <MUTATION> mutation?

© 2023 Benary M et al. *JAMA Network Open.*

eTable 4. Questions for the Survey

Area	Question
General	What is your profession?
	For how long have you been participating in molecular tumor boards?
Usefulness of treatment recommendation	Which of the presented options are in general useful? You can name specific treatment recommendations as well as complete options.
	Which of the presented clinical interpretations would you seriously consider for your decision?
Turing test	On a scale from 0 to 10, with 0 being not at all likely and 10 being extremely likely, how likely is it that one of presented set of recommendations is coming from an AI chatbot?
	Can you justify your decision in the question before?

© 2023 Benary M et al. *JAMA Network Open.*

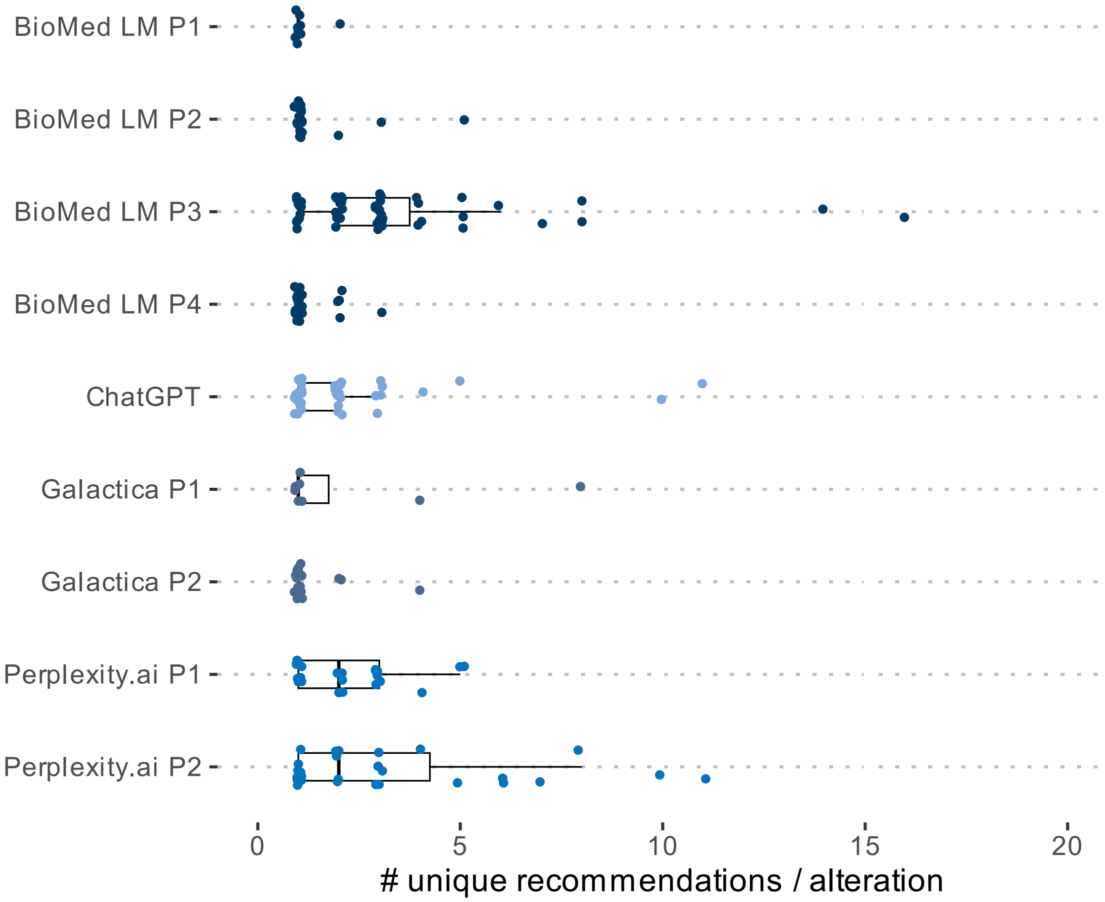

eFigure 1. Number of Treatment Options per Prompt Type

Each dot represents one alteration.

Recommendations by the LLMs are generated in two steps: First, prompting the LLM for treatment options given a patient case, and second, gathering the answers generated by the LLM and preparing for the evaluation. An illustration of this process is given in **Error! Reference source not found.**.

© 2023 Benary M et al. *JAMA Network Open.*

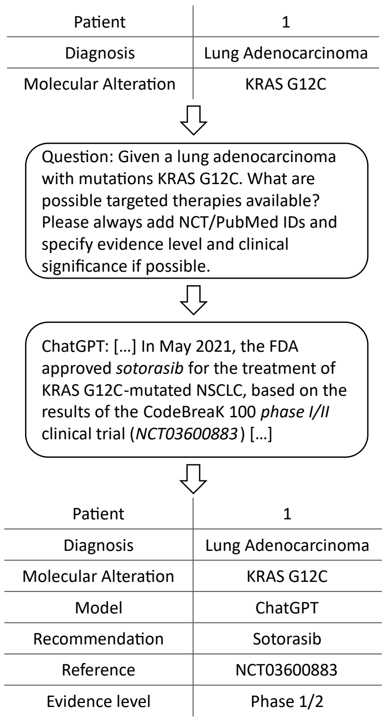

eFigure 2. Workflow of LLM Prompting

Convert the patient information into a natural language question, prompt the LLMs, and gather the outputs in a table.

© 2023 Benary M et al. *JAMA Network Open.*

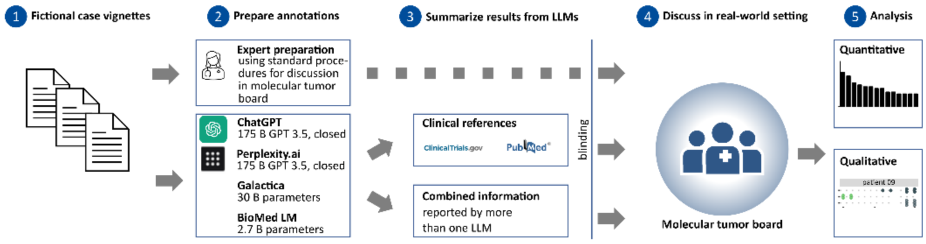

eFigure 3. General Workflow of the Analysis

Fictional case vignettes were provided to a human expert as well as four LLM for the identification of treatment options. Summarized LLM results and treatment options identified by the expert were blinded, presented to an interdisciplinary molecular tumor board and analyzed.

© 2023 Benary M et al. *JAMA Network Open.*

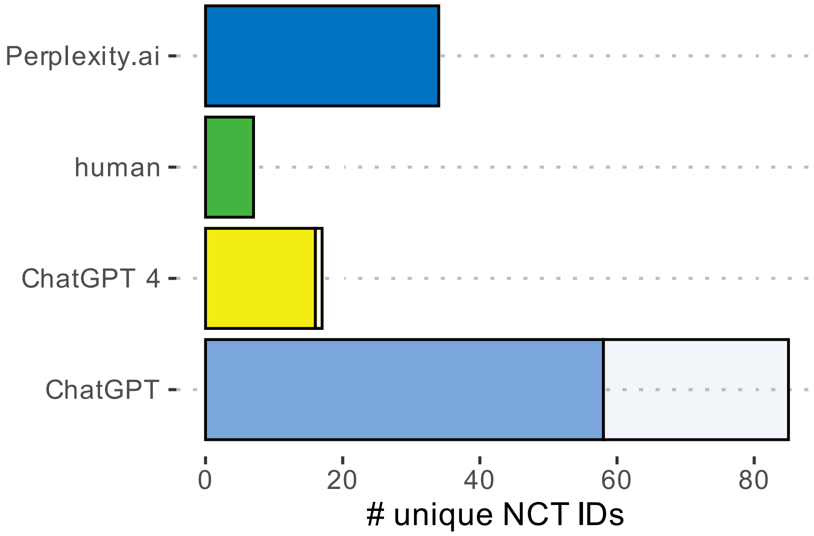

eFigure 4. Number of Unique Clinical Trials Suggested by LLMs and the Oncological Experts

Valid NCT studies are in a dark color, whereas hallucinations are depicted in a light color.

© 2023 Benary M et al. *JAMA Network Open.*

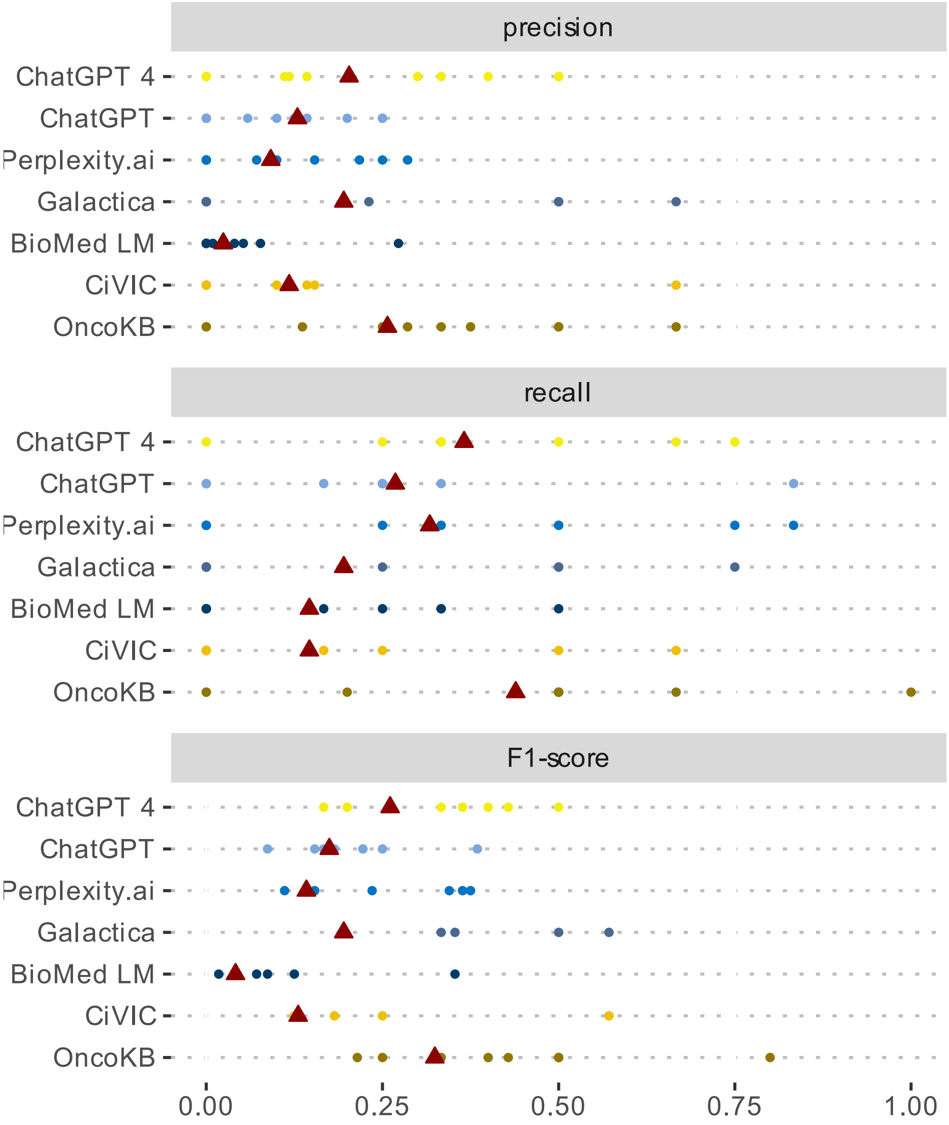

eFigure 5. Precision, Recall, and F1 Scores for the Structured Databases and LLMs Compared With the Human Criterion Standard

Scores for each patient are given in small dots and for all patients combined in triangles.

© 2023 Benary M et al. *JAMA Network Open.*

eReferences

1. Rieke DT, Lamping M, Schuh M, et al. Comparison of Treatment Recommendations by Molecular Tumor Boards Worldwide. *https://doi.org/101200/PO1800098*. 2018;(2):1-14. doi:10.1200/PO.18.00098

2. Brown T, Mann B, Ryder N, et al. Language Models are Few-Shot Learners. In: *Advances in Neural Information Processing Systems*. Vol 33. Curran Associates, Inc.; :1877-1901. Accessed October 11, 2022. https://papers.nips.cc/paper/2020/hash/1457c0d6bfcb4967418bfb8ac142f64a-Abstract.html

3. Ji Z, Lee N, Frieske R, et al. Survey of hallucination in natural language generation. *ACM Comput Surv*. 2023;55(12):1-38.

4. Griffith M, Spies NC, Krysiak K, et al. CIViC is a community knowledgebase for expert crowdsourcing the clinical interpretation of variants in cancer. *Nat Genet*. 2017;49(2):170-174. doi:10.1038/ng.3774

5. Chakravarty D, Gao J, Phillips S, et al. {OncoKB}: A Precision Oncology Knowledge Base. (1):1-16. doi:10.1200/PO.17.00011

6. Pallarz S, Benary M, Lamping M, et al. Comparative Analysis of Public Knowledge Bases for Precision Oncology. *JCO Precis Oncol*. 2019;(3):1-8. doi:10.1200/PO.18.00371

7. R Core Team. R: A Language and Environment for Statistical Computing. Published online 2021. https://www.r-project.org/

8. Gehlenborg N. UpSetR: A More Scalable Alternative to Venn and Euler Diagrams for Visualizing Intersecting Sets. Published online 2019. https://cran.r-project.org/package=UpSetR

© 2023 Benary M et al. *JAMA Network Open.*
