# Supplementary material for: Leveraging Large Language Models for Decision Support in Personalized Oncology
Source: JAMA Netw Open. 2023 Nov 17;6(11):e2343689. doi: 10.1001/jamanetworkopen.2023.43689 (PMC10656647; doi:10.1001/jamanetworkopen.2023.43689)
Supplement: Supplement 2. — Data Sharing Statement [file jamanetwopen-e2343689-s002.pdf]

Data Sharing Statement

Data
Data available: Yes
Data types: Deidentified participant data
How to access data: Data will be made available with supplementary files
When available: With publication

Supporting Documents
Document types: Statistical/analytic code
How to access documents: https://github.com/WangXII/LLMs_in_PO/
When available: With publication

Additional Information
Who can access the data: Data will be made available to anyone
Types of analyses: for non-profit purpose
Mechanisms of data availability: without investigator support
